# Shedding light on research participation effects in behaviour change trials: a qualitative study examining research participant experiences

**DOI:** 10.1186/s12889-016-2741-6

**Published:** 2016-01-29

**Authors:** Virginia MacNeill, Marian Foley, Alan Quirk, Jim McCambridge

**Affiliations:** 1 Faculty of Public Health & Policy, London School of Hygiene & Tropical Medicine, 15-17 Tavistock Place, London, WC1H 9SH UK; 2 Faculty Health, Psychology and Social Care Manchester Metropolitan University, 799 Wilmslow Road, Manchester, M20 2RR UK; 3 Royal College of Psychiatrists’ Centre for Quality Improvement, 21 Prescot Street, London, E1 8BB UK; 4 Department of Health Sciences, University of York, Heslington, York, YO10 5DD UK

**Keywords:** Behaviour change, Qualitative, Health promotion, Research participation effects, Hawthorne effect, Demand characteristics

## Abstract

**Background:**

The sequence of events in a behaviour change trial involves interactions between research participants and the trial process. Taking part in such a study has the potential to influence the behaviour of the participant, and if it does, this can engender bias in trial outcomes. Since participants’ experience has received scant attention, the aim of this study is thus to generate hypotheses about which aspects of the conduct of behaviour change trials might matter most to participants, and thus have potential to alter subsequent behaviours and bias trial outcomes

**Methods:**

Twenty participants were opportunistically screened for a health compromising behaviour (unhealthy diet, lack of exercise, smoking or alcohol consumption) and recruited if eligible. Semi structured face to face interviews were conducted, after going through the usual processes involved in trial recruitment, baseline assessment and randomisation. Participants were given information on the contents of an intervention or control condition in a behaviour change trial, which was not actually implemented. Three months later they returned to reflect on these experiences and whether they had any effect on their behaviour during the intervening period. Data from the latter interview were analysed thematically using a modified grounded theory approach.

**Results:**

The early processes of trial participation raised awareness of unhealthy behaviours, although most reported having had only fleeting intentions to change their behaviour as a result of taking part in this study, in the absence of interventions. However, careful examination of the accounts revealed evidence of subtle research participation effects, which varied according to the health behaviour, and its perceived social acceptability. Participants’ relationships with the research study were viewed as somewhat important in stimulating thinking about whether and how to make lifestyle changes.

**Conclusion:**

These participants described no dramatic impacts attributable to taking part in this study. This study demonstrates the likely value of well conducted qualitative studies of subtle research participation effects, which may be particularly important to explore for alcohol. Separating unintended influences in trial participation from the effects of behaviour change interventions being evaluated therein is necessary for valid estimates of intervention effects.

## Background

Behaviour change trials (or behavioural intervention trials) evaluate the effectiveness of interventions that seek to modify risky or unhealthy behaviours. These studies have interesting complexities. For example, participants may not themselves recognise that there is any need to change their behaviour, and ambivalence is common in relation to behaviours targeted for health promotion and public health purposes [1]. The processes involved in taking part in research studies also probably impact upon participants’ thinking and feelings about targeted behaviours [2] and it may also be the case that they influence the behaviours themselves. It is an obvious problem if the conduct of a study unwittingly impacts the study outcomes. In the context of randomised controlled trials, this suggests the possibility of biased estimates of intervention effects [3, 4]. Systematic reviews provide evidence that answering research questions, and engagement with other parts of the research process, can and do impact upon participants [5–8]. We know little, however, about the circumstances in which this is more likely to occur, and for whom, or how and why any such impacts occur.

Existing studies are mostly quantitative and designed to identify whether such effects exist. They largely lack important data regarding contextual factors that are relevant to these issues, with the exception of studies undertaken in particular contexts such as paediatric intensive care, where contextual effects might be expected to be pronounced [9, 10]. This particular limitation is best addressed using qualitative research methods, which are increasingly being viewed as important in trials. For example, Scott and colleagues [11] explored contextual effects not specific to the intervention under investigation in an acupuncture trial, and found that recruitment and retention were dependent on participants’ active involvement in a dynamic and contextually driven process.

Existing qualitative studies in this area tend to concentrate on certain aspects of study design, such as factors that influence recruitment [12]; relationships with the research staff [11] and unintended consequences arising from different levels of comprehension in relation to informed consent [13]. Other studies examine participant sense making processes in relation to trial identity [14], experiences of randomisation [15] and reasons for participation [16]. Wolters and colleagues [17] capture the experiences of participants longitudinally throughout the course of the study.

We are unaware of any qualitative studies that have been designed to elaborate the nature or possible extent of bias in quantitative study outcome data. Thus, as a preliminary study, we seek to find out what it might be like to be in a behaviour change trial from the perspective of potential participants, specifically in relation to possible pathways to impact on behaviour change outcomes. The aim of this study is thus to generate hypotheses about which aspects of the conduct of behaviour change trials might matter most to participants, and thus have potential to alter subsequent behaviours and bias trial outcomes [3].

## Methods

Twenty participants were opportunistically screened for health compromising behaviours (unhealthy diet, lack of exercise, smoking or alcohol consumption) and recruited to a research study by research assistant if eligible, by virtue of having one of more behavioural risk factor warranting preventive intervention in usual practice. Participants were thus not help-seeking, and though recruited in a busy London square, the process was otherwise designed to resemble how patients may be approached when attending, for example, their general practice. Data collection was carried out from late 2009 to early 2010. We do not have a record of how many people were approached, though all who were screened were eligible, and all were successfully recruited and retained. Study information material made clear that the study was designed to identify what research participants thought it might be like to be in a behavior change trial. Study participants were then telephoned the next working day, to confirm their interest and arrange an appointment to give consent to and take part in the first interview.

The first interview was conducted as if it was a randomised controlled trial, starting with consent, then a baseline assessment that addressed the behaviour(s) already identified during the screening process (smoking, drinking, unhealthy diet or lack of exercise). This trial did not take place. The assessment instruments used were drawn from those widely used in the relevant field of behavior change study [18–22]. These instruments were all designed for either screening or brief assessment purposes, and thus could be completed quickly. Participants were then randomised to either the control or intervention arm, using a sealed envelope procedure. A standard National Health Service information sheet describing recommended guidance on the relevant behaviours was distributed to all the participants. This simulated a not untypical control condition in these types of studies. Those notionally allocated to the intervention group were given an additional, more detailed information sheet that included a description of how participants might consult their GP or other professional for further support. Participants understood they were not expected to act on the information provided in both study conditions, that this was not a randomized controlled trial, and simply had to return for a second interview approximately three months later, when they would be asked to reflect on their experiences of this process (screening, study information provision, consent procedures, baseline data collection, the randomisation process and allocation to either control or intervention) and how it might affect people. When they did return, they repeated the baseline assessment as if it were a follow-up interview and they were then asked for their thoughts on the entire process of study participation. This qualitative study thus examines reflections on recruitment, the first interview and the first section of the follow-up interview, as they might be implemented in a trial, after which qualitative data collection commenced. We were particularly interested in whether there were any consequences during the preceding three months that they could attribute to what took place in recruitment or the first interview, or to study participation more generally.

The data we report are from the interviews undertaken face-to-face, 30–60 minutes long, audio-recorded, and subsequently transcribed verbatim. Interviews were conducted by a research assistant and followed an interview guide that was flexible enough to focus upon any issues brought up during the first interview. It specifically included topics such as recollection of the screening and randomisation processes, perceptions of personal health behaviours, and thoughts related to the intervention and control material, and possible impacts on their individual health behaviours. The transcribed data were organised with the aid of Nvivo10 software [23] and analysed thematically using some of the techniques of a grounded theory approach [24] including constant comparison [25, 26].

The approach taken was to initially open code the interview data case by case according to the main topics in the interview guide and then carry out a cross case analysis, separating the data into meaningful fragments and then systematically coding and labelling them to form an initial coding frame. The initial codes were discussed at regular team meetings and then developed into categories using both inductive and deductive approaches that were further refined into key themes. The interview data were analysed across the four different behavioural risk factors, which permitted triangulation to allow for a more comprehensive understanding of the topic.

The study was approved by the London School of Hygiene and Tropical Medicine Research (LSHTM) Ethics Committee (reference 5647).

## Results

The majority of participants were students or professionally qualified people (see participant details: Table 1 below). The mean age of the participants was 33 years old, and most were male (14 male, 6 female). All twenty participants returned for the second interview, approximately three months after the first.

**Table 1:** Participant details

ID	Placemet	Age	Sex	Occupation	Country of Origin^a^	Primary Behaviour	Secondary Behaviour, ^t^Randomisation outcome
1	London park	31	M	student	Italy	unhealthy diet	- intervention
2	London park	37	M	security controller	UK	smoking	risky drinking, intervention
3	London park	36	M	builder	UK	smoking	risky drinking, control
4	London park	30	M	public relations	UK	smoking	risky drinking, control
5	London park	28	F	PhD student	Germany	smoking	unhealthy diet, intervention
6	London park	47	M	academic researcher	UK	lack of exercise	-control
7	London pub	32	M	sales person	UK	risky drinking	-intervention
8	London park	25	F	student	UK	unhealthy diet	risky drinking, control
9	London park	19	M	student	UK	risky drinking	-control
10	London park	32	M	engineer	UK	Unhealthy diet	risky drinking, intervention
11	London park	45	M	company director	UK	smoking	risky drinking, intervention
12	London park	52	F	musician	UK	unhealthy diet	lack of exercise, control
13	London park	38	M	market researcher	UK	lack of exercise	unhealthy diet, intervention
14	London park	34	M	computer engineer	UK	unhealthy diet	risky drinking, control
15	London park	21	M	student	South Asia	unhealthy diet	-intervention
16	London park	28	F	youth worker	Poland	unhealthy diet	risky drinking, control
17	London park	28	F	archivist	UK	unhealthy diet	-control
18	London park	57	M	Door Manager	UK	unhealthy diet	risky drinking, control
19	London park	24	M	student	UK	risky drinking	-intervention
20	London park	24	F	student	Sweden	unhealthy diet	risky drinking, control

^a^Country of origin - observed/ learned through discussion during the interview. T In this paper secondary behaviours were disregarded as there was insufficient detail in the interview data as not the focus of the discussion

### Recalling the screening process and first interview

Participants reacted in different ways to the screening results. People with unhealthy diets and those undertaking little or no exercise said they were aware they needed to make changes. While a few participants spoke about guilty feelings when they made unhealthy eating choices, there was no suggestion that they felt any social repercussions. The research situation was seen as distinct from everyday life; eating was considered a normal activity that was not ordinarily discussed in the way it was in the research study:

***‘Because nobody ever says… well I had this for breakfast, I had this for lunch’*** (Unhealthy diet participant 12).

Similarly the non-exercisers acknowledged the need to become more active, typically labelling themselves as lazy. Neither group felt socially stigmatised by their behaviour, and they said that the onset of poor health or a personalised medical prognosis of poor health could motivate them to change.

Smoking and drinking were viewed somewhat differently. Smokers accepted the need to stop smoking to be more healthy, though the three risky drinkers did not, and reacted adversely to the notion that their alcohol consumption was excessive. Two of them stated that they needed to be convinced that their levels of consumption were sufficiently damaging to reduce drinking, preferring authoritative, personalized guidance on health impacts. For example, the participant below was doubtful about their level of risk or problems:

***‘Perhaps something I’ve written on the form is suggesting that I drink too much. So at that point you think, well, goodness, I didn’t think I drank that much. Perhaps my understanding of what’s acceptable is really, really wrong.’*** (Drinker participant 09***).***

It should be noted that screening for drinking alcohol, just like the other behaviours, identifies risk rather than provides evidence of existing health problems. Another was concerned about the possible implications of labelling:

***‘If you’re making an admission like that I’m an alcoholic, for instance, then that’s, that could come back and get you, couldn’t it? If it got into the wrong hands prevent you from getting a job or driving a motor vehicle, taxi driver, you’re not going to get a position like that. So perhaps it’s something you don’t necessarily want to put out into the wrong hat’*** (Drinker participant 19).

In contrast to the two drinkers above, the third drinker was ambivalent about whether he needed to cut down on his drinking:

***‘Sometimes I think it’s important and sometimes I don’t, sometimes I don’t think it’s enough of a problem to be putting the work in’*** (Drinker participant 07).

This person spoke of his shock previously at being told to give up by his doctor:

***‘It was the fact that a health professional saying to you, you should give up drinking, was more than the way he said, the way he said it was perfectly human, friendly and so on, and not particularly formal. It was more the, it was the fact that it was coming from him in that context, that was a shocker, yeah’*** (Drinker participant 07).

The randomisation process was well understood by the participants. Those who were allocated to the control arm were in some cases pleased because they believed it meant little further effort was required by involvement in the study:

***‘Glad that I wasn’t part of the extra work. I didn’t have to do anything else, it was more of a case of you were going to call me back in three months’ time, but from that time to that period I didn’t have to do anything. So I was quite happy with that’ (***Drinker participant 07).

Others were disappointed because they believed that allocation to the intervention arm would provide more help and motivation to change.

***‘I would like to be in the one that was being helped and more involved’*** (Unhealthy diet participant 14).

The written material handed out at the first interview, either standard patient information or describing an intervention, had limited impact on the participants as intended; nearly all said that they had not read it, or had forgotten about it within a day or so. Most participants were aware of, and broadly endorsed, the importance of a healthy lifestyle. Most reported accumulating knowledge about health from a variety of sources, but information *per se* was perceived as not enough to motivate any change. Particular kinds of information were seen as valuable, i.e. personalized information was viewed as being of direct benefit, and this was why they had so readily discarded the study information material. One to one discussions, reinforced by information that addressed their personal situation, were viewed as more likely to support change, but, again, one of the drinkers presented a different perspective when he said confronting excessive alcohol consumption did nothing but provoke feelings of guilt and difficulties about being honest.

### Views on behaviour change and the difficulties involved

Behaviour change was not regarded as a trivial undertaking. For some it was perceived as an isolating experience:

***‘When you’ve got an individual habit that needs to be eradicated, like giving up smoking, I think it’s like a very isolating situation that you are actually in. Because it’s just you as an individual, independently deciding at that particular moment in time, whatever period it is, that it’s time to give up and you’re not going through it with anyone else’*** (Smoker participant 02).

Nevertheless most of the participants reported leaving the first interview with some level of intention to make changes. This was transient, however, lasting no more than a day in most cases. By the second interview, three months later, only one participant reported that she had significantly changed her behaviour: she had stopped smoking (apart from the occasional cigarette).

While most participants recognized, to some degree, health benefits in modifying their lifestyles, they also discussed their health behaviours in the context of other pressures and demands on their time. Work was the most commonly cited barrier to change, perceived as having a negative impact on available time and energy levels; it meant having to prioritise in relation to other day to day activities. Life events were also seen as a distraction to acting on good intentions, and several participants described emotional situations where they had been unable to sustain any meaningful change. Even the very thought of giving up smoking or changing activity levels provoked anxiety and fear of failure in some. As several participants remarked, it was important to be in the right frame of mind, to be psychologically prepared, for behaviour change.

None of the drinkers reported change in drinking behavior itself, although they said that they had become more self-aware and questioning of their habits through taking part in the study. One participant pointed out that answering questions about drinking made him question his level of consumption, though as he did not suffer from any health problems at present, he would be unlikely to change his behaviour. Throughout the second interview, the participants vacillated between believing or not believing their health behaviours had changed and there were contradictory elements in some accounts. For example, one participant stated;

***‘It’s been no change really. It’s been much the same’*** (Unhealthy diet participant 12).

And elsewhere:

***‘I changed my diet, I’m not eating sugary things, or biscuits, not snacking at all, I’m walking more....’*** (Unhealthy diet participant 12).

### Perceptions of the research study as a catalyst for change

Few participants were able to clearly recall much about the first interview:

‘**Q*uestions about my diet but I can’t actually really remember what kind of questions that I was asked. I know it’s to do with fruit and veg intake and possibly lifestyle but I can’t really remember too much about that***’ (Unhealthy diet participant 17).

They reported giving the research study little more than an occasional thought, which was triggered by an event they associated with it, for example when passing the building where the first interview had taken place (LSHTM), or when they received a reminder about the second interview. While some could not recall specific details of the first interview, most reported having a grasp of the study aims:

***‘The line of questioning was about the way in which I respond as part of a study to the way that that study is constructed, so did I find a particular approach method effective or did I find the way that someone behaved towards me effective. It was the psychology of the approach, I suppose. That was the focus of what we talked about, I think*** ‘(Smoker participant 04).

***‘You were more trying to find out, it wasn’t about my wellbeing or my health, it was more probably the mind-set of trying to understand about getting people to do these sort of surveys or studies. So it’s probably just more for you to get an understanding of how you can get more people involved with studies. If my memory serves me right it wasn’t necessarily about trying to give me advice on how to improve my fitness or my health’*** (Non exercise  participant 06).

***Most reported that their participation in the study had some impact on their thinking, attitudes, or awareness of their lifestyle:***

***‘Any conversation you have about something that you know is bad that you do anyway, particularly when you’re forced directly to talk about it for an hour solid, you can’t help but that’s, that’s floating around in there. That’s going to be, whether I like it or not, that’s going to be something that’s there for a couple of weeks after it’s left, so, yeah, subconsciously, definitely’*** (Smoker participant 04***).***

Some participants in all four behaviour groups reported, in some cases without great certainty, that the study had some impact on their thinking about their own behavior, and that this was a possible prelude to behaviour change:

***‘Possibly being involved in this has slightly increased the number of moments, where I’ve thought I really must make that change tomorrow. Tomorrow doesn’t come, but that’s about it’*** (Non exercise participant 13***).***

***‘I just thought about it more than I normally would and that’s, it hasn’t completely transformed my drinking or anything but it’s made me think just a bit and will hopefully probably lead to me drinking [less]’.*** (Drinker participant 19).

***‘It makes me think almost directly after these sessions and then it dips a little bit but it’s made me think now about certain things that’s for sure. So, yeah, it’s definitely brought out a few things in the foreground now that, yeah, I will do, there’s things that I will think about, just not buying cookies anymore’*** (Unhealthy diet participant 14).

***‘Well maybe, I don’t know, but I was thinking about it before, but I’ve started thinking again about that I probably really should stop, quit smoking, but I don’t know if it, this study made any difference to my thinking’*** (Smoker participant 05).

One participant suggested that further contacts between researchers and participants might make more of a difference to participants:

***I think I’m more guided by my own common sense and what I observe rather than what I found out in this study. However, I think had this study been more intense, as in if I, if I’d been seeing you every week or so and, or participated in a study that is much more intense and in depth I think it would have had more impact’*** (Unhealthy diet participant 16).

## Discussion

This study was undertaken to gain a sense of the possible value of qualitative data in better understanding unintended consequences of research participation; to advance our appreciation of what participants can tell us about the effects of taking part in a trial and what looks most promising to study. These participants described no dramatic impacts attributable to taking part in this study (in being recruited, in the first interview and in the initial section of the second interview) and also revealed how complex it was to gain a sense of whether their own behaviour may have changed in small ways. It was suggested that there may be small and subtle impacts not directly upon particular behaviours, but rather on thinking about these issues. Recall of earlier research participation events was patchy. Various challenges involved in actually changing behaviours were articulated clearly. Among the four behaviours covered here, alcohol consumption stood out as being particularly sensitive.

Process studies are increasingly being implemented within trials to address how studies actually take place and how interventions may exerts their effects [27]. Unintended impacts of the research study on participant behaviour and other impacts would seem very appropriate to be included in these types of studies, and we have made a start in this direction by adding a brief set of questions to process study interviews and gained valuable data in so doing [28]. We suggest this study provides proof of concept, that there are issues that can be usefully explored by asking participants about them. Interviews can yield experiential data that speak to important and not well understood concerns relating to the effects of taking part in these and other types of research studies. Participants are far from passive in research studies, and the possible impacts of taking part are not likely to be discernible in research assessments until we know much better what we’re looking to find out. Interviews with participants should help with that [29]. The study design used here is useful because it isolates research participation effects from the effects of intervention.

### Limitations

The study design has clear limitations, because the conceptual generalizability of the findings to actual behavior change trials is unknown. As a result, we consider the limitations of this study in some detail.

We were broadly encouraged that these were authentic and credible accounts, gained without being unduly influenced by trying to please us, and that it is indeed possible to collect the types of data we sought by interviewing trial participants. Both the internal and external validity of these findings, however, warrant careful consideration in light of the nature of the data. Participants felt comfortable telling us that this study had mattered little to them. It is impossible to assess the veracity of accounts of possible subtle impacts on thinking about changing healthy behaviours, though these accounts do appear plausible, being consistent with the available literature. The construct of demand characteristics [7] offers one conceptualisation in this context; has our construction of this research context cued participants to provide us with certain types of data, and not others. We did not draw attention to areas where the literature is strongest, for example on the effects of answering questions [4–6, 8], in ways which led participants to tell us what they might have thought we wanted to hear. Given that our study focus was on unintended reactivity in research, we were well placed to be mindful of the risks of evoking particular types of reactivity.

Whilst these observations can be offered in support of the assessment of internal validity, external validity is more challenging to consider. Our sample was somewhat highly educated, being recruited in the vicinity of various universities, and though having more men than women and a relatively narrow age band, was otherwise fairly unremarkable in relation to the general population. This study was also not nested within an ongoing behavior change trial. If research participation effects are strongly contextually driven [29], then our artificial research context will have little generalizability. On the other hand, if research participation effects are primarily artefacts of the research process (being screened, assessed, randomized etc.) then a scrupulous concern for what participants have to say about these processes may be more widely relevant across contexts [29]. We don’t know which of these two possibilities is more likely.

### Hypotheses generated and implications for further study

It appears likely on the basis of these data, that existing behaviour change models, such as the stages of change [30], may aid thinking about how impactful or not taking part in this research may be. Motivational accounts of behaviour change emphasise that it is a process not an event [31]. The evidence of study participation promoting thinking about change could be described as prompting movement from pre-contemplation to contemplation. It is not clear, however, where, if anywhere, this may lead. It is possible that such stimuli will help prepare people to become more receptive to interventions, though this seems more likely for behaviours they have not thought about changing before [3, 5].

People who are less inclined to consider changing their behaviour will be by definition more challenging to intervene with. They also may well be less likely to participate in the first place, and less likely to be retained over time in studies that rely on opportunistic recruitment [32]. These considerations suggest it is important to consider carefully the likely levels of motivation for behaviour change both in terms of designing interventions, but also in the design of recruitment to trials evaluating the effects of such interventions, and in measures to avoid loss to follow-up. For example, one randomised study found the salience of requested outcome data for participants influenced rates of attrition [33]. The present study findings are in line with those of Wolters and colleagues [17], who suggest that the activities of research assistants and their engagement with participants may be particularly worthy of further study.

Differences among the behaviours were striking, and social desirability considerations were prominent. It seems likely that the potential for research participation effects will vary across behaviours, and alcohol may be particularly interesting to further explore in this regard. This behaviour was seen here as challenging to intervene with, and the apparent gap between the intentions of researchers interested in alcohol and participant understanding of this behaviour may itself generate research participation effects. In the context of everyday lives, any such effects compete with a range of other influences on thinking about health and behavior change, in ways that suggest the effects will be small. Just as the effects of interventions to change health behaviours are small, this makes imperative the need for better understanding and control of reactivity effects in order to produce valid inferences about intervention effectiveness.

## Conclusion

This study demonstrates the likely value of well conducted qualitative studies of research participation effects, and indeed in health behaviour change trials. Although more direct effects upon behaviour are worthy of investigation in studies nested within behaviour change trials, attention to more subtle impacts of particular research procedures, on thoughts and feelings about behaviour change, appears important to pursue. Such studies should be designed to appreciate the active engagement of participants with the specific contexts we offer them in behaviour change trials and other types of research studies. Separating the influences of participation in trials from the behaviour change interventions evaluated therein is both challenging and necessary.
